# Supplementary material for: Identifying virulence determinants of multidrug-resistant Klebsiella pneumoniae in Galleria mellonella
Source: Pathog Dis. 2021 Jan 29;79(3):ftab009. doi: 10.1093/femspd/ftab009 (PMC7981267; doi:10.1093/femspd/ftab009)
Supplement: ftab009_Supplemental_Files [file ftab009_supplemental_files.zip › Galleria_Supplement.docx]

# Identifying virulence determinants of multidrug-resistant *Klebsiella pneumoniae* in *Galleria mellonella*

## Supplementary tables

**Table S1: TraDIS sequencing information**

| **Strain** | **Sample** | **Sample Acc** | **Lane 1** | **Lane 2** | **Run 1** | **Run 2** | **Sample Read Count** | **Transposon Reads** | **Mapped Reads** | **Unique Insertions** |
| --- | --- | --- | --- | --- | --- | --- | --- | --- | --- | --- |
| RH201207 | *G. mellonella* infection Input 1 | ERS2856142 | 24686_1#10 | 24686_2#10 | ERR3313543 | ERR3313564 | 14,671,625 | 14,611,926 | 13,067,937 | 426,116 |
| RH201207 | *G. mellonella* infection Input 2 | ERS2856143 | 24686_1#11 | 24686_2#11 | ERR3313544 | ERR3313565 | 15,139,931 | 15,075,494 | 13,489,139 | 432,678 |
| RH201207 | *G. mellonella* infection Input 3 | ERS2856144 | 24686_1#12 | 24686_2#12 | ERR3313545 | ERR3313566 | 13,907,983 | 13,848,936 | 12,399,651 | 426,248 |
| RH201207 | *G. mellonella* infection 2 hpi 1 | ERS2856148 | 24686_1#16 | 24686_2#16 | ERR3313549 | ERR3313570 | 14,008,792 | 13,952,271 | 12,471,099 | 254,076 |
| RH201207 | *G. mellonella* infection 2 hpi 2 | ERS2856149 | 24686_1#17 | 24686_2#17 | ERR3313550 | ERR3313571 | 14,494,416 | 14,430,166 | 12,887,292 | 256,592 |
| RH201207 | *G. mellonella* infection 2 hpi 3 | ERS2856150 | 24686_1#18 | 24686_2#18 | ERR3313551 | ERR3313572 | 14,543,107 | 14,481,084 | 12,959,253 | 256,600 |
| RH201207 | *G. mellonella* infection 6 hpi 1 | ERS2856151 | 24686_1#19 | 24686_2#19 | ERR3313552 | ERR3313573 | 14,752,537 | 14,691,081 | 13,174,812 | 254,756 |
| RH201207 | *G. mellonella* infection 6 hpi 2 | ERS2856152 | 24686_1#20 | 24686_2#20 | ERR3313553 | ERR3313574 | 14,053,700 | 13,996,625 | 12,594,311 | 211,200 |
| RH201207 | *G. mellonella* infection 6 hpi 3 | ERS2856153 | 24686_1#21 | 24686_2#21 | ERR3313554 | ERR3313575 | 13,031,469 | 12,975,166 | 11,619,148 | 233,170 |
| ATCC 43816 | *G. mellonella* infection Input 1 | ERS2856117 | 23257_1#1 | 23363_1#1 | ERR4706232 | ERR4706238 | 1,966,025 | 1,959,181 | 1,901,817 | 301,651 |
| ATCC 43816 | *G. mellonella* infection Input 2 | ERS2856118 | 23257_1#2 | 23363_1#2 | ERR4706233 | ERR4706239 | 1,858,337 | 1,851,902 | 1,796,586 | 301,089 |
| ATCC 43816 | *G. mellonella* infection Input 3 | ERS2856119 | 23257_1#3 | 23363_1#3 | ERR4706234 | ERR4706240 | 2,003,569 | 1,996,407 | 1,937,781 | 307,248 |
| ATCC 43816 | *G. mellonella* infection 4 hpi 1 | ERS2856120 | 23257_1#4 | 23363_1#4 | ERR4706235 | ERR4706241 | 2,085,144 | 2,076,503 | 2,001,084 | 234,563 |
| ATCC 43816 | *G. mellonella* infection 4 hpi 2 | ERS2856121 | 23257_1#5 | 23363_1#5 | ERR4706236 | ERR4706242 | 1,962,113 | 1,953,683 | 1,882,388 | 227,021 |
| ATCC 43816 | *G. mellonella* infection 4 hpi 3 | ERS2856122 | 23257_1#6 | 23363_1#6 | ERR4706237 | ERR4706243 | 1,945,318 | 1,938,289 | 1,875,950 | 232,356 |

**Table S2: Plasmid replicons in RH201207 identified by PlasmidFinder**

| **Plasmid** | **Identity** | **Query length / Template length** | **Contig** | **Position in contig** | **Accession number** |
| --- | --- | --- | --- | --- | --- |
| ColRNAI | 100 % | 130 / 130 | RH201207_8 | 10359-10488 | DQ298019 |
| IncFIB(K) | 100 % | 560 / 560 | RH201207_7 | 4026-4585 | JN233704 |
| IncFIB(pQil) | 100 % | 740 / 740 | RH201207_2 | 217-956 | JN233705 |
| IncFII(K) | 97.8 % | 148 / 148 | RH201207_2 | 50275-50422 | CP000648 |
| IncFII(K) | 97.8 % | 148 / 148 | RH201207_6 | 1728-1875 | CP000648 |
| IncX3 | 100 % | 374 / 374 | RH201207_5 | 32841-33214 | JN247852 |

**Table S3: Unique insertion sites of the TraDIS libraries used**

| **Sample** | **Chromosome length** | **Unique Insertion Sites (UIS)** | **Length / UIS** |
| --- | --- | --- | --- |
| RH201207 | 5,475,790 bp | 510,834 | 10.72 bp |
| RH201207 2 hpi | 5,475,790 bp | 379,343 | 14.43 bp |
| RH201207 6 hpi | 5,475,790 bp | 359,602 | 15.23 bp |
| ATCC 43816 | 5,374,835 bp | 415,608 | 12.93 bp |
| ATCC 43816 4 hpi | 5,374,835 bp | 350,363 | 15.34 bp |

**Table S4: Pearson correlation coefficient *r*^2^ values of the comparisons of biological replicates by reads per gene, insertion indices per gene and reads per unique insertion site (UIS), respectively.**

| **Sample** | **Reads per gene** | | | **Insertion index per gene** | | | **Reads per UIS** | | |
| --- | --- | --- | --- | --- | --- | --- | --- | --- | --- |
|  | **rep 1 vs**  **rep 2** | **rep 2 vs**  **rep 3** | **rep1 vs**  **rep 3** | **rep 1 vs**  **rep 2** | **rep 2 vs**  **rep 3** | **rep1 vs**  **rep 3** | **rep 1 vs**  **rep 2** | **rep 2 vs**  **rep 3** | **rep1 vs**  **rep 3** |
| **RH201207 input** | 0.9988 | 0.9988 | 0.9976 | 0.9943 | 0.9945 | 0.9944 | 0.9589 | 0.959 | 0.9548 |
| **RH201207 2 hpi** | 0.948 | 0.9957 | 0.995 | 0.983 | 0.984 | 0.9831 | 0.7692 | 0.769 | 0.7704 |
| **RH201207 6 hpi** | 0.9922 | 0.9938 | 0.9938 | 0.9801 | 0.9792 | 0.9828 | 0.7078 | 0.6929 | 0.7337 |
| **ATCC 43816 input** | 0.9983 | 0.9982 | 0.9979 | 0.9845 | 0.9838 | 0.9852 | 0.9627 | 0.9644 | 0.9628 |
| **ATCC 43816 4 hpi** | 0.9959 | 0.9941 | 0.9924 | 0.9764 | 0.9765 | 0.9772 | 0.9145 | 0.913 | 0.9151 |

## Supplementary figures


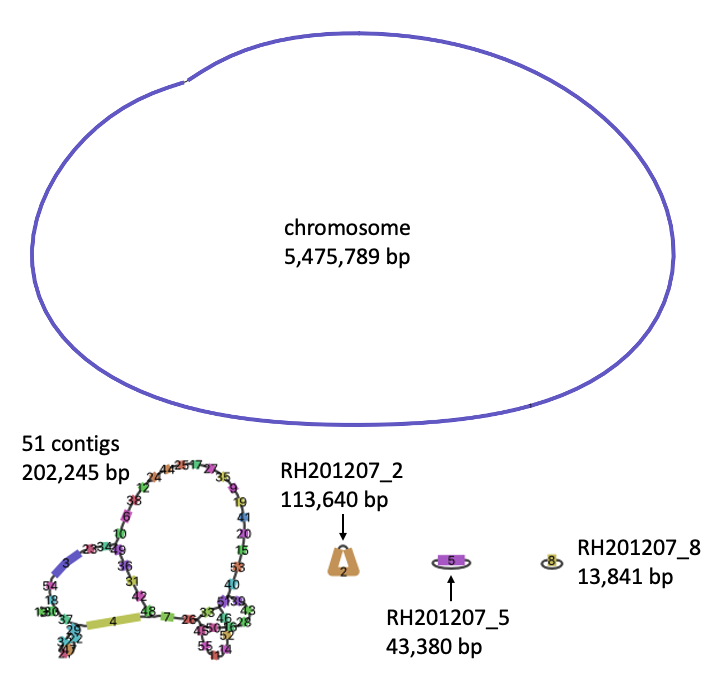


**Figure S1: Assembly graph of the RH201207 genome.** The genome was assembled using long Nanopore and short Illumina reads using Unicycler (Wick *et al.* 2017). This graphical representation of the assembly was generated with Bandage (Wick *et al.* 2015) and shows a circularised chromosome and three circularised plasmids (RH201207_2, RH201207_5 and RH201207_8). One structure, with a length of 202,245 bp consisting of 51 contigs and probably representing one or multiple plasmids could not be fully resolved.


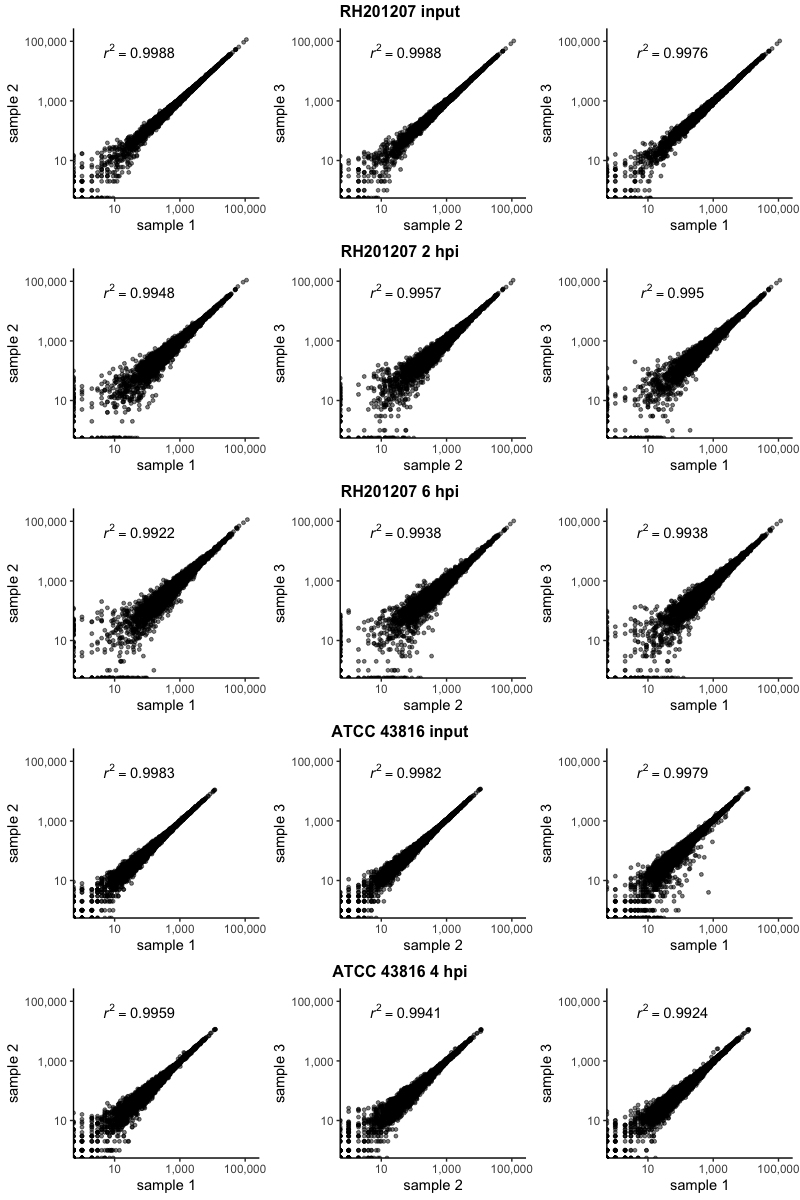


**Figure S2: Pairwise correlation of reads per gene of biological TraDIS replicates and their Pearson correlation coefficients**


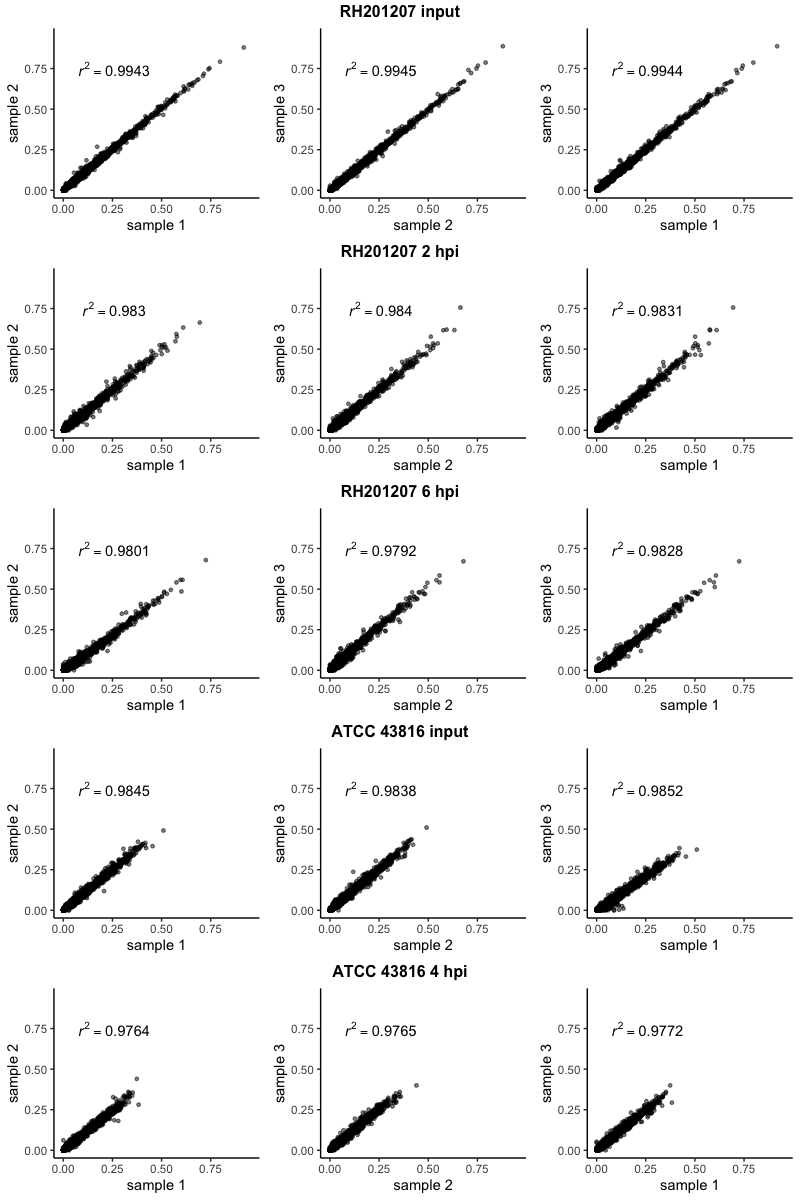


**Figure S3: Pairwise correlation of insertion indices per gene of biological TraDIS replicates and their Pearson correlation coefficients**


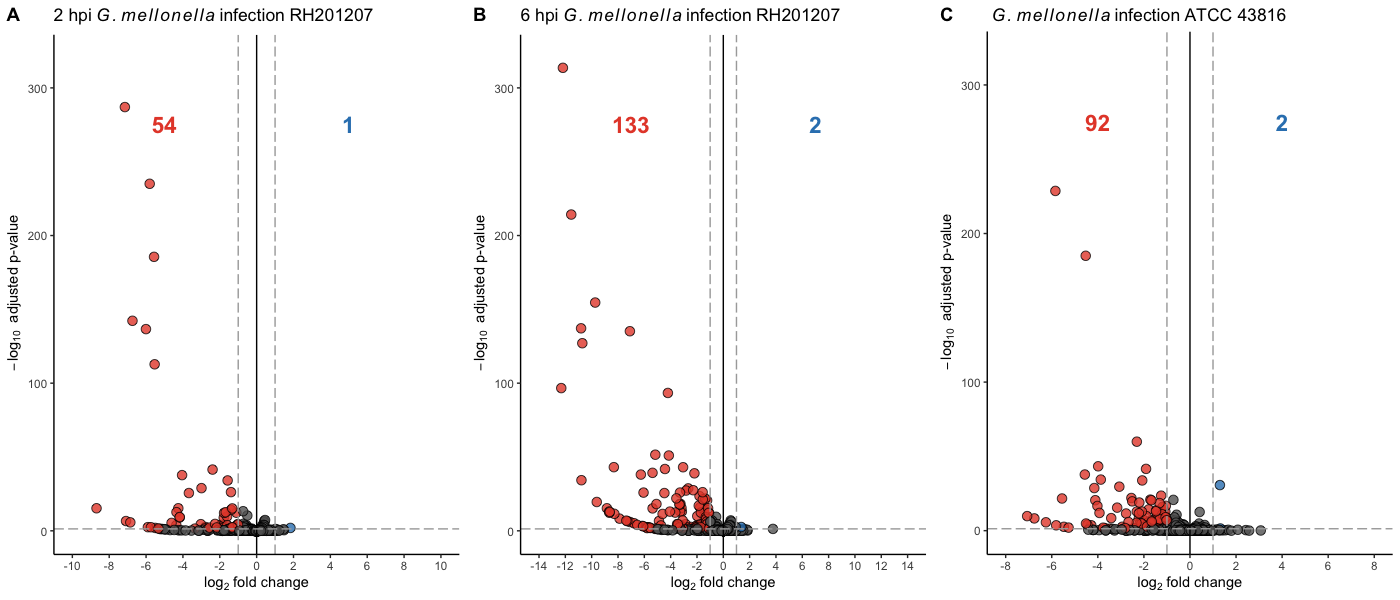


**Figure S4: Volcano plots of TraDIS datasets.** Significantly less abundant genes after infecting *G. mellonella* larvae are shown in red and significantly higher abundant genes in blue. Non-significant genes are shown in grey. Genes were considered significant if they possessed a Benjamini-Hochberg corrected *p*-value below a threshold of 0.05 and an absolute log2 fold-change greater than 1. Essential and ambiguous-essential genes are removed from the analysis. A: RH201207 at 2 hpi, B: RH201207 at 6 hpi, C: ATCC 43816 at 4 hpi.


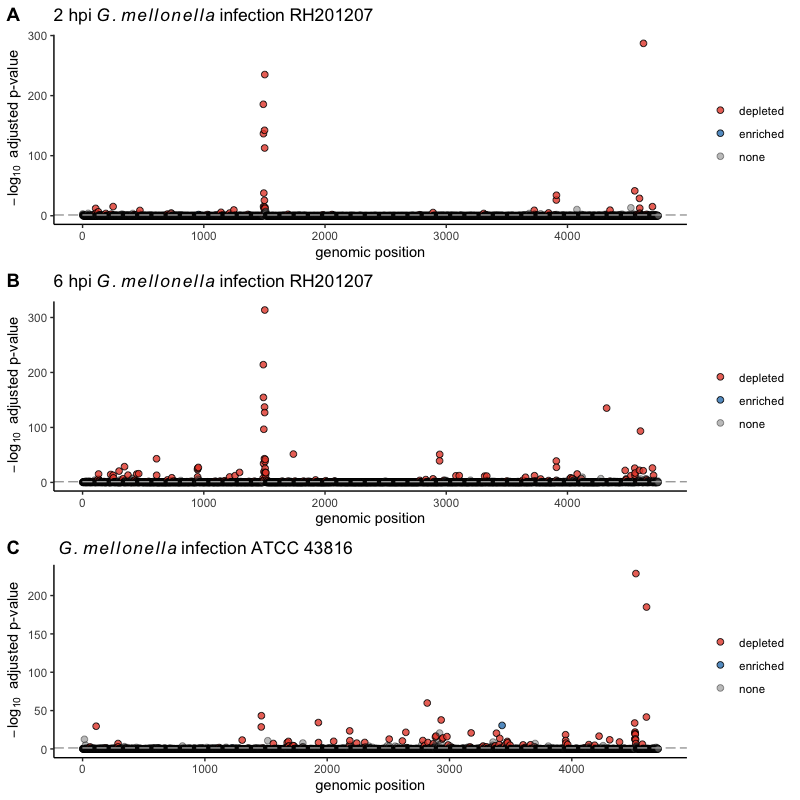


**Figure S5: Manhattan plots of TraDIS datasets showing adjusted p-value vs genomic position.** Significantly less abundant genes after infecting *G. mellonella* larvae are shown in red and significantly higher abundant genes in blue. Non-significant genes are shown in grey. Genes were considered significant if they possessed a Benjamini-Hochberg corrected *p*-value below a threshold of 0.05 and an absolute log2 fold-change greater than 1. Essential and ambiguous-essential genes are removed from the analysis. A: RH201207 at 2 hpi, B: RH201207 at 6 hpi, C: ATCC 43816 at 4 hpi.

**
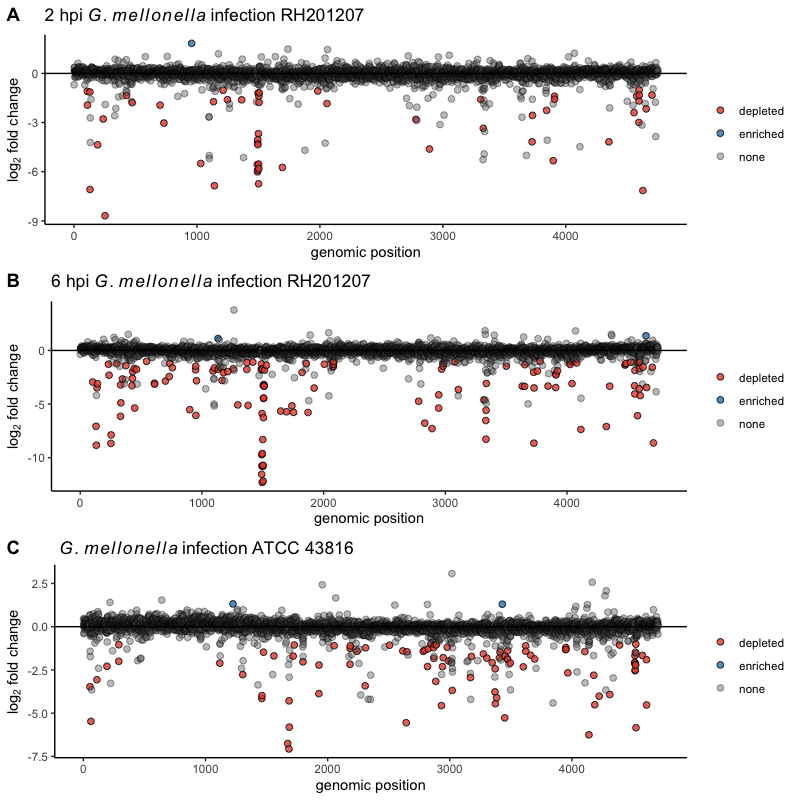
**

**Figure S6: Manhattan plots of TraDIS datasets showing log2 fold-change vs genomic position.** Significantly less abundant genes after infecting *G. mellonella* larvae are shown in red and significantly higher abundant genes in blue. Non-significant genes are shown in grey. Genes were considered significant if they possessed a Benjamini-Hochberg corrected *p*-value below a threshold of 0.05 and an absolute log2 fold-change greater than 1. Essential and ambiguous-essential genes are removed from the analysis. A: RH201207 at 2 hpi, B: RH201207 at 6 hpi, C: ATCC 43816 at 4 hpi.

## References

Broberg CA, Wu W, Cavalcoli JD *et al.* Complete Genome Sequence of Klebsiella pneumoniae Strain ATCC 43816 KPPR1, a Rifampin-Resistant Mutant Commonly Used in Animal, Genetic, and Molecular Biology Studies. *Genome Announc* 2014;**2**, DOI: 10.1128/genomeA.00924-14.

Demarre G, Guérout A-M, Matsumoto-Mashimo C *et al.* A new family of mobilizable suicide plasmids based on broad host range R388 plasmid (IncW) and RP4 plasmid (IncPα) conjugative machineries and their cognate Escherichia coli host strains. *Research in Microbiology* 2005;**156**:245–55.

Dorman MJ, Feltwell T, Goulding DA *et al.* The Capsule Regulatory Network of Klebsiella pneumoniae Defined by density-TraDISort. *mBio* 2018;**9**, DOI: 10.1128/mBio.01863-18.

Jana B, Cain AK, Doerrler WT *et al.* The secondary resistome of multidrug-resistant Klebsiella pneumoniae. *Sci Rep* 2017;**7**:42483.

Poulter S, Carlton TM, Spring DR *et al.* The Serratia LuxR family regulator CarR 39006 activates transcription independently of cognate quorum sensing signals. *Mol Microbiol* 2011;**80**:1120–31.

Short FL, Di Sario G, Reichmann NT *et al.* Genomic Profiling Reveals Distinct Routes To Complement Resistance in Klebsiella pneumoniae. *Infect Immun* 2020;**88**, DOI: 10.1128/IAI.00043-20.

Wick RR, Judd LM, Gorrie CL *et al.* Unicycler: Resolving bacterial genome assemblies from short and long sequencing reads. Phillippy AM (ed.). *PLoS Comput Biol* 2017;**13**:e1005595.

Wick RR, Schultz MB, Zobel J *et al.* Bandage: interactive visualization of de novo genome assemblies. *Bioinformatics* 2015;**31**:3350–2.
